# Spatiotemporal dynamics and potential ecological drivers of acute respiratory infectious diseases: an example of scarlet fever in Sichuan Province

**DOI:** 10.1186/s12889-022-14469-y

**Published:** 2022-11-21

**Authors:** Cheng Li, Rujun Liao, Wenhui Zhu, Guiyu Jiang, Yumeng Wang, Ling Li, Tao Zhang, Qiang Lv

**Affiliations:** 1 Department of Epidemiology and Health Statistics, West China School of Public Health and West China Fourth Hospital, Sichuan University, Chengdu, Sichuan China; 2 Sichuan Center for Disease Control and Prevention, Chengdu, Sichuan China

**Keywords:** Spatiotemporal analysis, Potential ecological drivers, Scarlet fever, Acute respiratory infections

## Abstract

**Object:**

Scarlet fever is an acute respiratory infectious disease that endangers public health and imposes a huge economic burden. In this paper, we systematically studied its spatial and temporal evolution and explore its potential ecological drivers. The goal of this research is to provide a reference for analysis based on surveillance data of scarlet fever and other acute respiratory infectious illnesses, and offer suggestions for prevention and control.

**Method:**

This research is based on a spatiotemporal multivariate model (Endemic-Epidemic model). Firstly, we described the epidemiology status of the scarlet fever epidemic in Sichuan Province from 2016 to 2019. Secondly, we used spatial autocorrelation analysis to understand the spatial pattern. Thirdly, we applied the endemic-epidemic model to analyze the spatiotemporal dynamics by quantitatively decomposing cases into endemic, autoregressive, and spatiotemporal components. Finally, we explored potential ecological drivers that could influence the spread of scarlet fever.

**Results:**

From 2016 to 2019, the incidence of scarlet fever in Sichuan Province varied much among cities. In terms of temporal distribution, there were 1–2 epidemic peaks per year, and they were mainly concentrated from April to June and October to December. In terms of transmission, the endemic and temporal spread were predominant. Our findings imply that the school holiday could help to reduce the spread of scarlet fever, and a standard increase in Gross Domestic Product (GDP) was associated with 2.6 folds contributions to the epidemic among cities.

**Conclusion:**

Scarlet fever outbreaks are more susceptible to previous cases, as temporal spread accounted for major transmission in many areas in Sichuan Province. The school holidays and GDP can influence the spread of infectious diseases. Given that covariates could not fully explain heterogeneity, adding random effects was essential to improve accuracy. Paying attention to critical populations and hotspots, as well as understanding potential drivers, is recommended for acute respiratory infections such as scarlet fever. For example, our study reveals GDP is positively associated with spatial spread, indicating we should consider GDP as an important factor when analyzing the potential drivers of acute infectious disease.

**Supplementary Information:**

The online version contains supplementary material available at 10.1186/s12889-022-14469-y.

## Background

Acute respiratory infection (ARI) is one of the leading causes of morbidity and mortality worldwide. Every year, approximately 156 million instances of acute respiratory infections are reported, with children under the age of 5 and those over the age of 65 being the most vulnerable [1]. According to the estimate of the World Health Organization (WHO) [2], ARIs rank as the fourth-highest global cause of mortality and resulting in nearly 3 million deaths worldwide in 2016 (40 deaths per 100,000). Acute respiratory infections such as scarlet fever [3], Ebola [4], and coronavirus disease 2019 (COVID-19) [5] have historically posed a substantial threat to public health and imposed a significant economic burden.

While it is critical to have heightened vigilance regarding emerging high-risk respiratory infections, we should not lose sight of those re-emerging infectious diseases with high incidence and outbreaks. A typical example of emerging infectious diseases is COVID-19, which caused a heavy burden on public health and the economy. As of 1 July 2022, there have been 545,226,550 confirmed cases of COVID-19, including 6,334,728 deaths [6]. Another typical example of the re-surged traditional infectious disease is scarlet fever. It was associated with high levels of morbidity and mortality when epidemics were common in the 18th and 19th centuries in Europe and the USA [7]. Although this disease nearly disappeared during the twentieth century, many countries have recently experienced a re-emergence of scarlet fever. This global re-emergence of scarlet fever has caused more than 600,000 cases around the world [8]. Scarlet fever is re-emerging as a public health threat.

However, developing vaccines, viral mutations, and the growing problem of antibiotic-resistant bacteria pose remarkable challenges in the prevention of ARIs such as scarlet fever [9]. Therefore, investigations on epidemiology and spatiotemporal dynamics are still critically needed to offer the scientific foundation for disease control and prevention. The spatiotemporal studies of infectious diseases often based on surveillance data and easily accessible variables. At present, there are two types of methods for modeling infectious disease surveillance data [10]: one is the mechanistic model, and the other is the statistical model. Mechanistic models, such as SIR models, build on extensive knowledge of basic epidemiology and aim to capture the important mechanisms of disease transmission. Statistical models can be used to explore and explain statistical variability in data. But the former approach ignores the dynamics of space [11], and the latter (statistical models) could not capture specific characteristics of infectious disease [12].

In order to solve the above problems and to better explore the infectious disease on surveillance data, Held [13] et al. proposed a spatio-temporal model (also known as Epidemic-Epidemic Model, EE model) on the basis of branching process, combining the mechanistic model and statistical model. This model decomposes the disease incidence into an “endemic” and an “epidemic” part (e.g, epidemic with region and epidemic among regions), which can simulate the spread of infectious disease and quantify the effect of covariates in different components. This modeling framework has been used widely to analyze other diseases such as tuberculosis [14], influenza [15], dengue [16], and so on.

As for the spatiotemporal study of scarlet fever, most studies concentrated on dynamics of the epidemic part (e.g, epidemic from temporal aspect and epidemic from spatial aspect) [17–20], ignoring the endemic part (e.g, indirect transmission from the environment). And also they did not qualify the effect of covariates in different dimensions (i.e, time, space, and endemic). In recent years, more and more study shows ecological factors could be a strong force in transmission [21]. However, previous research on scarlet fever focused on meteorological factors and failed to explore the association between ecological factors and spread [18, 19, 22]. To better understand the spatiotemporal dynamics of scarlet fever and explore potential ecological drivers in different dimensions, we applied the EE model to visualize space-time dynamics and describe the factors related to transmission.

Most spatiotemporal research of ARIs is based on the surveillance data of infectious diseases and a limited number of covariates, the urgency of the analysis, the accessibility of covariates, and the result of instability are the main challenges. In this paper, we took scarlet fever as an example, using the EE model to decompose the transmission into three dimensions: endemic, epidemic within city, and epidemic among cities. We also explore the effects of ecological factors in these dimensions. We hope our study can provide a reference for scarlet fever and other ARIs.

## Method

### Data

#### Case report

According to Chinese Infectious Diseases Law, when doctors identify any probable, clinical, or laboratory-confirmed case of scarlet fever, they need to report the case to the Notifiable Infectious Diseases Reporting Information System (NIDRIS) within 24 h [23].

#### Data collection

The data of reported cases (including the patient’s age, sex, occupation, and address) of scarlet fever were collected by the Nationwide Notifiable Infectious Diseases Reporting Information System (NIDRIS), which was used under license and not publicly available. We extracted data on scarlet fever in 2016–2019 of the Sichuan province from the NIDRIS, and the data included the number of cases, and patient data on age, sex, and date of disease onset, diagnosis, and address limited to cities. Because all the data in our study were anonymous and without access to the identity information of the patients, and hence, informed consent were waived by the ethics committee of the Sichuan Center for Disease Control and Prevention. The research has been approved by the ethics committee of the Sichuan Center for Disease Control and Prevention (SCCDCIRB-2022-185).

Additionally, we collected data on ecological factors from the *Sichuan Statistical Yearbook* (http://tjj.sc.gov.cn/scstjj/c105855/nj.shtml), which included population, population density, number of health institutions, number of primary schools, number of kindergartens, passenger-kilometers of Highways and Gross Domestic Product (GDP).

#### Study area

Sichuan province, located in the hinterland of southwest China, between 26°03′ -34 °19′ N and 97°21′ -108 °33′ E, is located in the upper reaches of the Yangtze River and covers an area of 486,000 km^2^, ranking fifth in China. Sichuan consists of 21 cities and prefectures, with the fourth largest population and the sixth GDP in China. The development of social and economic conditions varies between cities and prefectures in the province.

### Statistical analysis

#### Descriptive statistics

We cleaned and analyzed the data using R version 4.0.2. Count data were expressed by frequency and incidence. Firstly, we described the characteristics of population distribution and spatiotemporal distribution of scarlet fever. Secondly, we applied Global Moran’s *I* statistics to calculate the spatial autocorrelation [24]. The formula is defined as:

1 $$\begin{array}{l}I=\frac{n\sum_{i,j}\omega_{ij\left(X_i-\overline X\right)\left(X_j-\overline X\right)}}{\sum_{i,j}\omega_{ij}\sum_{i=1}^n\left(X_i-\overline X\right)^2}\\\end{array}$$

where n denotes the number of spatial units, *ωij* denotes the weight matrix, *X* denotes the variable of interest (i.e, incidence), and $\overline{X}$ denotes the mean value of all units. The value for Moran’s *I* can range from − 1 to 1 where:

i) 1: The variable of interest is perfectly dispersed;
ii) 0: The variable of interest is randomly dispersed;
iii) 1: The variable of interest is perfectly clustered together.

Thirdly, we used Anselin’s Local Moran’ *I* (local indicators of spatial association, LISA) test statistics to explore the clusters or outliers in the study area. For the spatial unit *i*, the formula is defined as [25]:

2 $$Ii=\frac{X_i-\overline X}{S^2}\sum_j\omega_{ij}\left(X_{\mathrm j}-\overline X\right)$$

where *S*^2^ denotes the variance, the value of *I*_*i*_ can disclose spatial clusters or outliers. There are five categories according to the value: high-high, low-low, high-low, low-high, and non-significant. The high-high and low-low areas represent spatial clusters, while the high-low and low-high areas were the outliers. The spatial analysis was conducted by GeoDa 1.20.

#### Transmission decomposition

We adopted the Endemic-epidemic model developed by Held and Paul [26] to decompose the incidence level. The formulas are as below:

3 $${\displaystyle \begin{array}{l}{Y}_{i,t}\mid {Y}_{i,t-1}\sim NegBin\left({\mu}_{it},\psi \right)\\ {}{\mu}_{it}={\nu}_{it}{e}_{it}+{\lambda}_{it}{Y}_{i,t-1}+{\phi}_{it}\sum \limits_{j\ne i}{\omega}_{ij}{Y}_{i,t-1}\end{array}}$$

Infectious disease counts *Y*_*i*, *t*_ in the region *i* = 1,…,21 during weeks *t* = 1,…208. *Y*_*i*, *t*_ follows a negative binomial distribution with the mean *μ*_*it*_ and overdispersion parameter *ψ* > 0. We decomposed the transmission into three component as follows:

1. endemic component (*ν*_*it*_*e*_*it*_): infections from outside the study area or from indirect transmission (i.e, infected by bacteria from environment)
2. autogressive component (*λ*_*it*_*Y*_*i*, *t* − 1_): reproduction of scarlet fever within city *i* (i.e,epidemic within city)
3. neighborhood component (${\phi}_{it}\sum \limits_{j\ne i}{\omega}_{ij}{Y}_{i,t-1}$): transmission from other regions except city *i* (i.e,epidemic among cities).

In Eq. (3), *ν*_*it*_ is log-linear predictor of the endemic component that, multiplied by an offset such as population *e*_*it*_, could describe incidence due to sociodemographic variation. The coefficient *λ*_*it*_ represents the transmission of infections from the past period, and the coefficient *ϕ*_*it*_ quantifies the contribution of spatial transmission capturing infections from other cities. We defined the spatial weight matrix as

4 $${\omega}_{ij}={O}_{ij}^{-\rho }$$

where *O*_*ij*_ represents the path distance between cities *j* and *i*, and *ρ* is the decay parameter can be estimated from the data. The path distance *O*_*ij*_ is on an ordinal scale based on the adjacency index [27].

Meanwhile, we added seasonal variation and covariates related to ecological situation as follows:

5 $$\left\{\begin{array}{l}\log\left(\nu_{it}\right)=\alpha_0+\alpha_i+\kappa_{it}^T\alpha+\sum_S\gamma_s\sin\left(\theta t\right)+\delta_s\cos\left(\theta t\right)\\\log\left(\lambda_{it}\right)=\beta_0+\beta_i+u_{it}^T\beta\\\log\left(\phi_{it}\right)=\gamma_0+\gamma_i+Z_{it}^T\gamma\end{array}\right.$$

6 $$\gamma_s\sin\left(\theta t\right)+\delta_s\cos\left(\theta t\right)=As\sin\left(\theta t+\varphi\right)$$

7 $$As=\sqrt{\gamma_s^2+{\delta}_s^2}$$

Where *α*_0_, *β*_0_, and *γ*_0_ are intercepts; ${\kappa}_{it}^T$, ${u}_{it}^T$ and ${Z}_{it}^T$ are covariates. Due to the covarites in our study could not explain the spatiotemporal heterogenity, we introduce random effects *α*_*i*_, *β*_*i*_ and *γ*_*i*_ with ${\alpha}_i\sim N\left(0,{\sigma}_{\alpha}^2\right)$, ${\beta}_i\sim N\left(0,{\sigma}_{\beta}^2\right)$, ${\gamma}_i\sim N\left(0,{\sigma}_{\gamma}^2\right)$ in the model; *α*, *β*, *γ* denotes the coefficient of covariates. Seasonal terms $\left\{\sum \limits_S{\gamma}_s\sin \left(\theta t\right)+{\delta}_s\cos \left(\theta t\right)\right\}$ reflect seasonally varying incidence [28], and *A*_*s*_ is the amplitude of the corresponding sine-cosine curve and $\theta =\frac{2\pi }{52}$.

According to previous research [27], the power-law algorithm was superior to other methods for spatial weight. Relevant parameter estimation was based on penalized maximum likelihood ratio method [29]. Given that the Akaike information criterion (AIC) is inappropriate to compare models that include random effects [30], we used the ranked probability scores (RPS) and logS scores (logS) [31] to select the best model and quantify the effect of covariates among the three components. These scores measure differences between the predicted distribution *P* of the fitted model and the observed value y. The lower the score is, the better the fit. We first chose the best-fixed effect model by AIC, then we added the random effects and use logS and RPS to evaluate the final model. The model framework is based on the R package “surveillance” [32]. We also used the packages including “spdep”, “sf” , “ggplot2” and “dplyr” to run endemic-epidemic model.

#### Exploration of ecological effects

As an ecological study, we also aim to provide clues from the perspective of correlation between covariates and the spread of infectious diseases. For the analysis of surveillance data, indicators of ecological factors are easy to gather. We took scarlet fever in Sichuan as an example to explore the effects of ecological factors on three components of transmission. However, as with most, this study could not include all factors, so we introduced random effects in our model to alleviate the uncertainty caused by covariates.

Our model incorporated multiple ecological covariates. Table A1 in the Additional file describes the meaning and values of covariates that could influence the spread of the infectious disease [33–37]. We added the covariates in three components based on epidemiology knowledge. First, we use the stepwise regression method to explore the optimal fixed effect model by AIC. We began with an intercept-only model (model 1) with a population offset in the endemic component and spatial weight matrix based on the power law. Secondly, we sequentially added ecological covariates in the three components. Thirdly, we added random effects to capture more realism in our model. Last, we chose the final model by comparing logS and RPS.

## Results

### Basic characteristic

Table 1 summarizes the basic characteristic of patients. A total of 7356 scarlet fever cases were reported from 2016 to 2019 in Sichuan province. Males outnumbered females. (4388 vs. 2968 cases). The youngest patient was confirmed 1 day after birth, and the oldest patient is 59 years old. The majority of recorded cases (7160 cases, 97.34%) came from the native population rather than the floating population (i.e, a large and increasing number of migrants without local household registration status). The incidence of the 4–6 years group was the highest of all age groups (Table A2 in Additional file), which is more than 1 per 100,000 population each year.

**Table 1:** Description of scarlet fever cases in Sichuan Province from 2016 to 2019

		Year			
	Total N(%)	2016 N(%)	2017 N(%)	2018 N(%)	2019 N(%)
Gender					
Female	2968 (40.35)	657 (38.47)	805 (40.21)	754 (41.27)	752 (41.34)
Male	4388 (59.65)	1051 (61.53)	1197 (59.79)	1073 (58.73)	1067 (58.66)
Age (year)					
0–3	1287 (17.50)	306 (17.92)	356 (17.78)	298 (16.31)	327 (17.98)
4–6	3890 (52.88)	916 (53.63)	1096 (54.75)	1003 (54.90)	875 (48.10)
7–9	1670 (22.70)	390 (22.82)	453 (22.62)	399 (21.84)	428 (23.53)
10–12	296 (4.03)	64 (3.75)	72 (3.60)	86 (4.71)	74 (4.07)
13–15	106 (1.44)	15 (0.88)	16 (0.80)	19 (1.04)	56 (3.08)
16–69	107 (1.45)	17 (1.00)	9 (0.45)	22 (1.20)	59 (3.24)
Source of cases					
Other province	57 (0.77)	11 (0.64)	16 (0.80)	11(0.61)	19 (1.04)
Foreign country	1 (0.01)	1 (0.06)	0 (0.00)	0 (0.00)	0 (0.00)
The same city	7160 (97.34)	1675 (98.07)	1933 (96.55)	1728 (97.53)	1770 (97.30)
Other cities	138 (1.88)	21 (1.23)	53 (2.65)	34 (1.86)	30 (1.66)
Total	7356	1708	2002	1827	1819

**Fig. 1:**
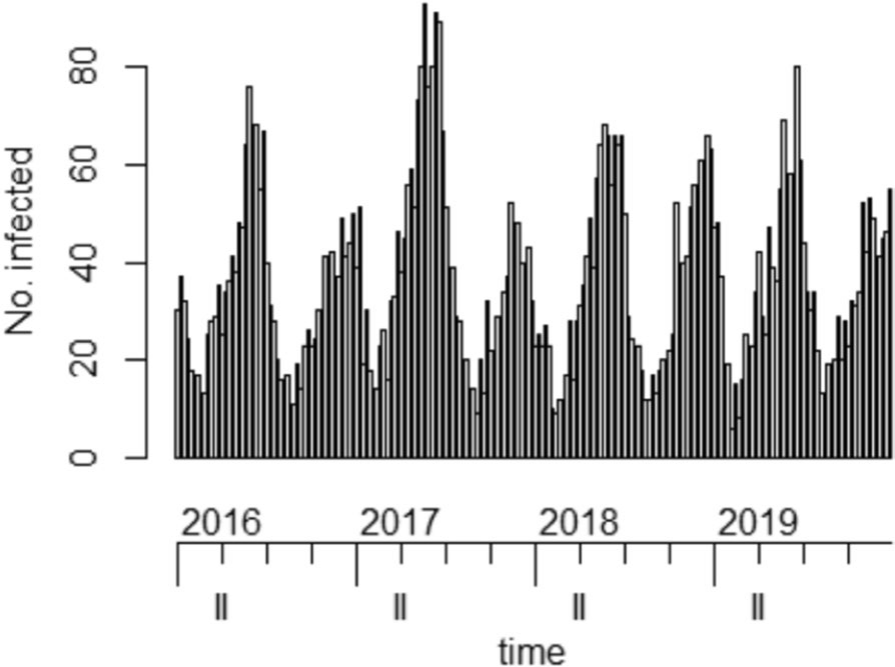
Time series of scarlet fever counts in Sichuan province from 2016 to 2019

Figure 1 shows the temporal distribution of Sichuan province from 2016 to 2019. It also can be seen that there is a certain seasonality and periodicity between 2016 and 2019, with 1–2 epidemic peaks every year. The peak period of incidence was mainly concentrated in the second quarter of the year (April to June). A few areas occasionally have a small peak period in the fourth quarter (October to December), such as Mianyang in 2018 (Fig. A1 in Additional file).

Scarlet fever cases were found in 21 cities and prefectures across Sichuan province, with the number of cases varying in different cities (Fig. A1 in Additional file). To be specific, Chengdu ranked first, with a total of 2428 cases, accounting for about one-third of the total cases. Leshan, Mianyang, and Liangshan were the following three cities (/autonomous prefectures). Ya ‘an and Ganzi had the fewest number of cases, with no more than 10 cumulative cases in the 4 years. The annual average incidence was 1.868 cases (per 100,000 population).

**Fig. 2:**
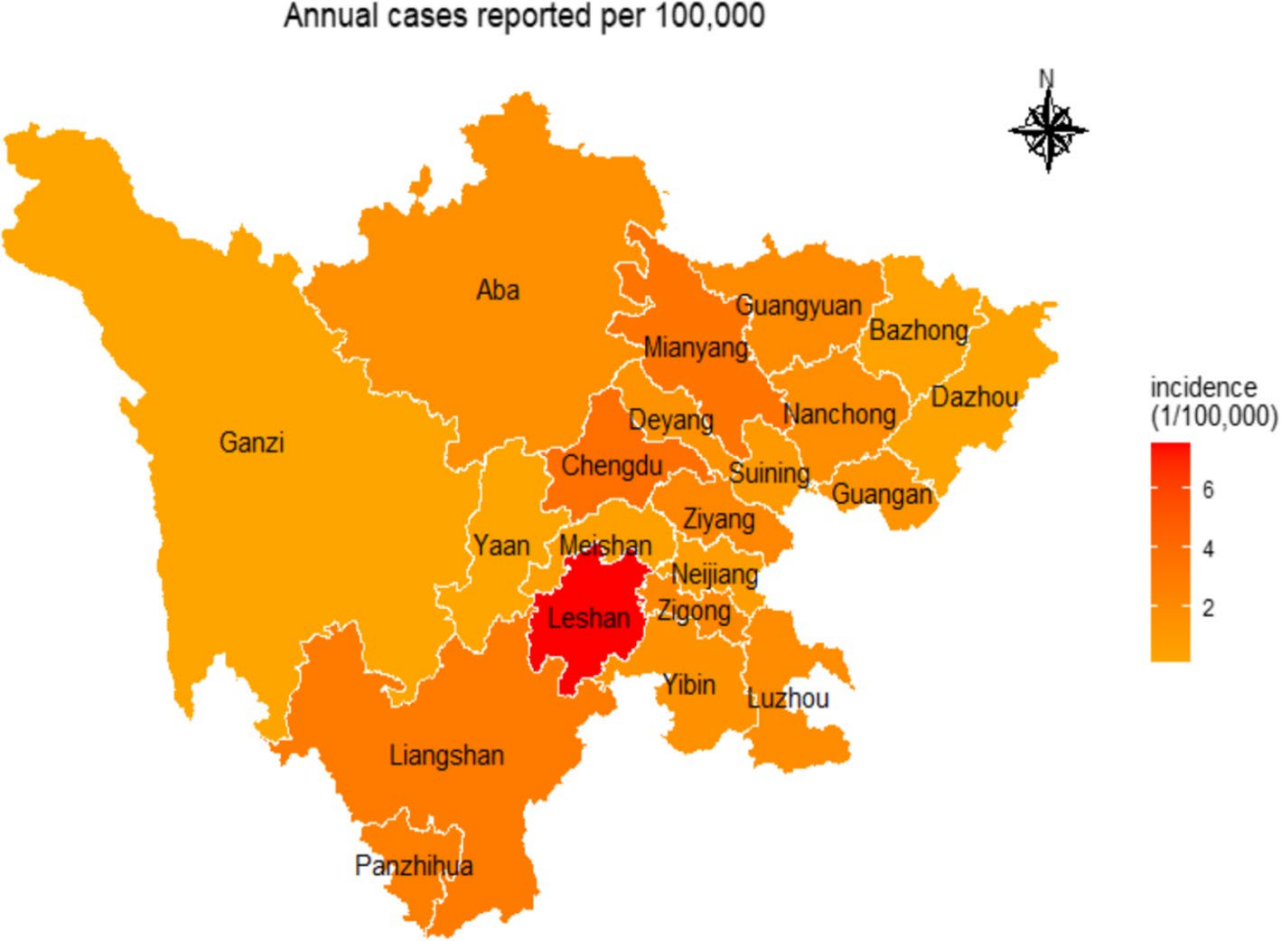
The average incidence of scarlet fever in Sichuan province from 2016 to 2019

Figure 2 shows the spatial distribution. The incidence of scarlet fever varies greatly in different areas. Cases were mainly concentrated in Leshan, Mianyang, Chengdu, and Aba Tibetan Autonomous Prefecture. Among them, Leshan had the highest average annual incidence of 7.556 cases (/100,000 population), whereas Ganzi Tibetan Autonomous Prefecture had the lowest average annual incidence rate of 0.0825 cases (/100,000 population).

**Fig. 3:**
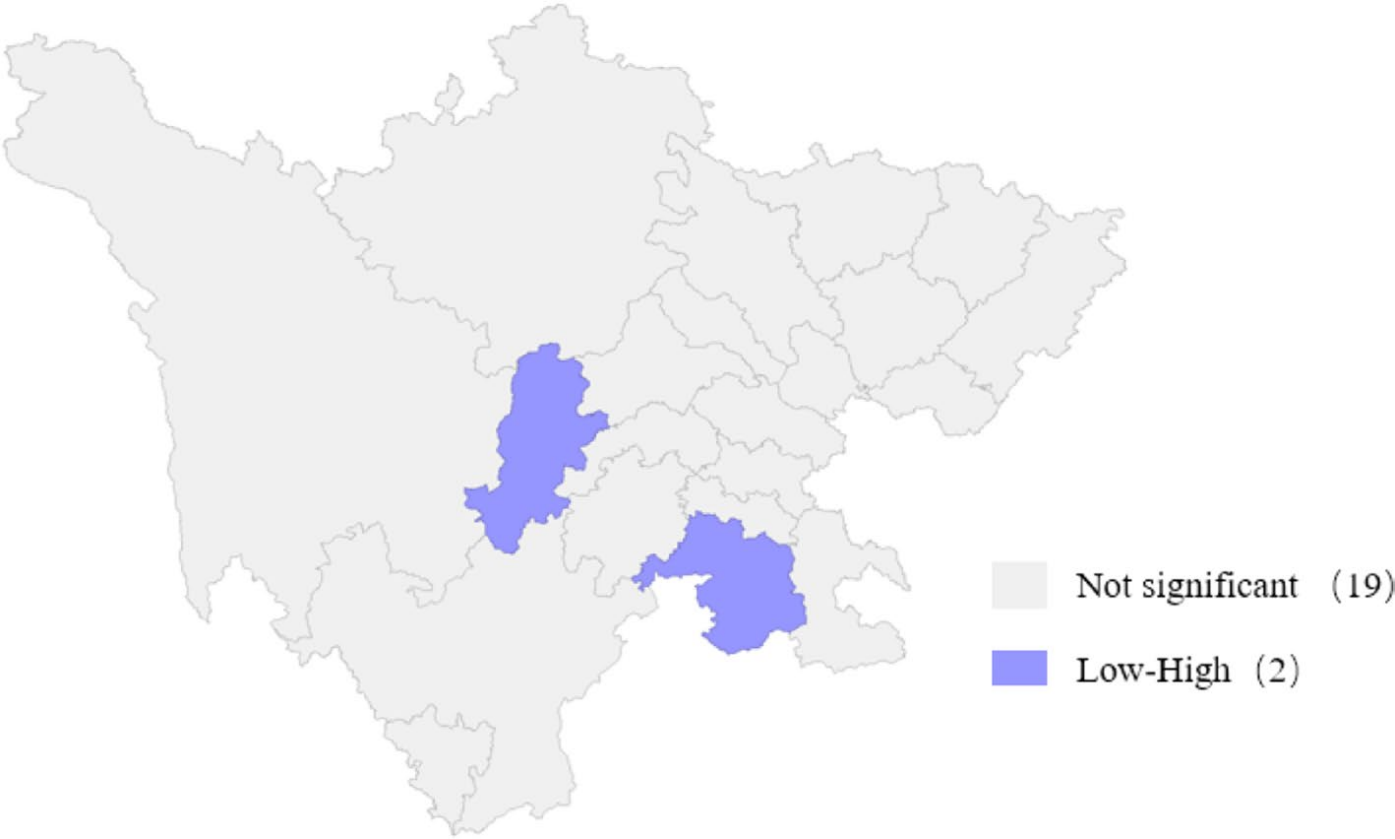
LISA map of scarlet fever in Sichuan province during 2016–2019. The light blue parts indicate two spatial outliers, one is Ya’an, and the other is Yibin

In our study, the global Moran’s *I* = -0.0512 (*Z* = -0.013, *P* = 0.484), indicated no significant spatial autocorrelation of scarlet fever incidence during 2016–2019 in Sichuan province. For Local Moran’s *I*, as shown in Fig. 3, there were only two low-high outliers with light blue: Ya’an and Yibin, where the given cities evidenced low incidences but were surrounded by high incidence areas.

### Transmission decomposition

**Fig. 4:**
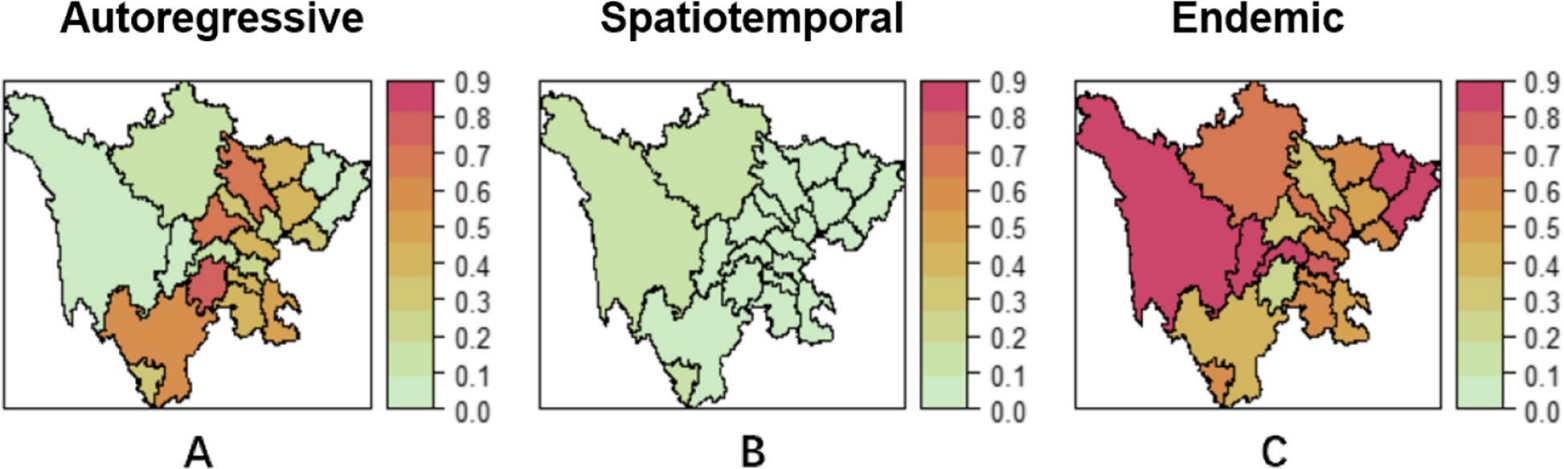
Maps of the fitted component proportions averaged all over weeks. **A** Autoregressive components, **B** Spatiotemporal component, **C** Endemic component

Figure 4 shows the averaged proportions of fitted components all over weeks. It can be seen that the autoregressive component and endemic component have a certain proportion, whereas the proportion of the spatiotemporal component is very low. This is consistent with the results spatial autocorrelation, which means scarlet fever is less affected by spatiotemporal transmission. According to the average proportions shown in Fig. 4, we classified three types of transmission as follows:

1. The main transmission is resulted from endemic, including Ganzi, Aba, Ya’an, Meishan, Dazhou, and Bazhong.
2. The main transmission is epidemic within city, including Mianyang, Chengdu, Leshan, and Liangshan.
3. The main transmission are resulted from epidemic within city and endemic, including Ziyang, Neijiang, Zigong, Yibin, Luzhou, Suining, Nanchong, Guang’an, Guangyuan, Deyang, and Panzhihua.

**Fig. 5:**
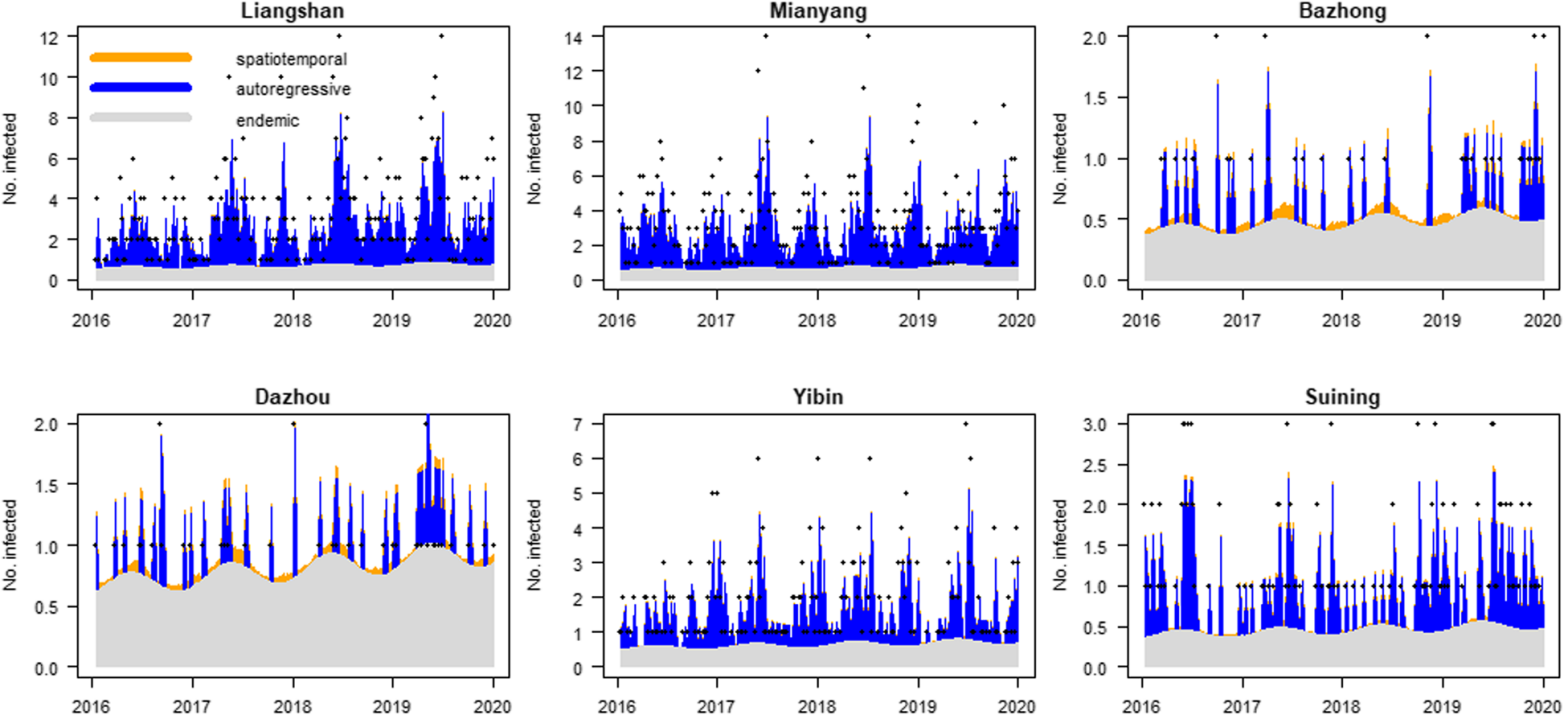
Fitted components in the endemic-epidemic model for the typical six cities. The plots were divided into three components: endemic component, autoregressive component (epidemic within city) and spatiotemporal component (epidemic among cities). The black dots represent the number of observed cases, the orange part represents spatial transmission, the blue part represents temporal transmission, and the grey part represents the endemic component

Fig. A2 (Additional file) presented fitted values for 21 cities (/prefectures) and Fig. A3 in the Additional file reveals the seasonality of the endemic mean. We took the six cities of Liangshan, Mianyang, Dazhou, Bazhong, Yinbin, and Suijing as examples (Fig. 5). The incidence was quite low in Dazhou and Bazhong, and the transmission was predominantly composed of endemic and a little spatiotemporal component. In high-incidence cities such as Liangshan and Mianyang, scarlet fever cases were mainly influenced by transmitted cases from previous periods. In Suining and Yibin, the transmission was composed of endemic and autoregressive components

### Association with ecological factors

Table A3 in the Additional file presents a summary of the model selection and comparison process. The model 5 yielded the lowest AIC (from 11903 in model 1 to 11,604.41 in model 5 without random effects, in Additional file, Table A3). In the final model, the autoregressive component included population density, school holidays, GDP, number of health institutions, number of preschools, and number of kindergartens. The spatiotemporal component consisted of population density, GDP, and passenger kilometers of Highway. Endemic component comprised school holidays, number of preschools and number of kindergartens.

Because ecological factors could not fully explain the spatiotemporal variation of transmission mechanisms and incidence levels, we allowed the intercept (mean levels of *ν*_*it*_, *λ*_*it*_ and *ϕ*_*it*_) in the three components of infections to vary for each city as random effects. The results of the final model were shown in Table 2. Table 2 shows the relative risk (RR) and 95% confidence interval (CI) for each explanatory variable in different components. The overdispersion parameter is 0.1053, which means our data of surveillance is discrete. The weight decay parameter is 0.0108, indicating there is a low correlation among cities. Fig. A4 and Fig. A5 in the Additional file shows the spatial connectivity weights and weight matrix, respectively. School holidays is a protective factor in both endemic component and autoregressive component (RR = 0.4712, 95%CI: 0.2776–0.7999; RR = 0.5299, 95%CI: 0.3883–0.7233). With each standard level increase in GDP, the contribution to the spatial transmission was higher by 2.6 folds (RR = 2.6898, 95% CI: 1.1516–6.2822).

**Table 2:** Coefficient estimates from the endemic-epidemic model

Parameter	RR	95%CI	*P* value
Epidemic within the city (autoregressive)			
Intercept	0.1588	(0.0034, 7.4853)	–
Density	0.8162	(0.5192, 1.2831)	0.379
School holidays	**0.4712**	(0.2776, 0.7999)	< 0.001
GDP	1.6331	(0.9146, 2.9160)	0.097
number of health institutions	0.8328	(0.2562, 2.7073)	0.761
number of kindergartens	1.0954	(0.4324, 2.7752)	0.848
number of primary schools	0.7951	(0.3146, 2.0093)	0.628
Epidemic among cities (spatiotemporal)			
Intercept	0.0024	(0.0001, 2.5915)	–
Density	1.1721	(0.6524, 2.1058)	0.595
GDP	**2.6898**	(1.1516, 6.2822)	0.022
Passenger-kilometers of highways	0.7528	(0.4971, 1.1400)	0.180
Endemic			
Intercept	0.9965	(0.9929, 1.0001)	–
School holidays	**0.5299**	(0.3883, 0.7233)	< 0.001
number of kindergartens	2.1440	(0.6288, 7.3111)	0.223
number of primary schools	0.4996	(0.1780, 1.4021)	0.188

Statistically significant covariates are in bold

### Random effects

**Fig. 6:**
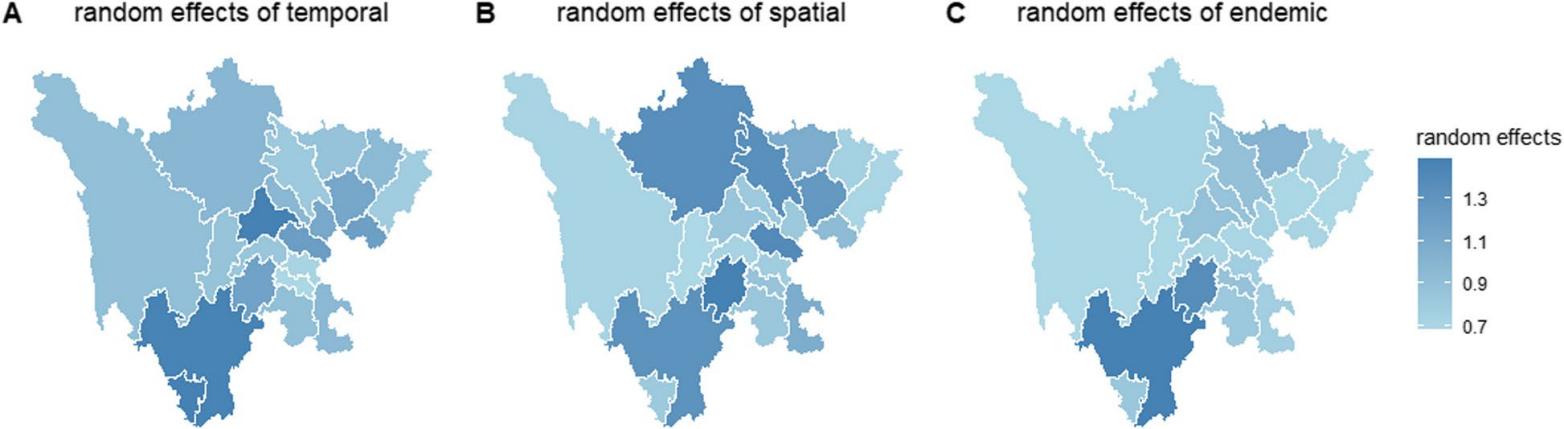
Random effects for three components of the model. **A** Random effects of epidemic within city component; **B** Random effects of epidemic between cities component. **C** Random effects of endemic component

Random effects are useful if the regions exhibit heterogeneous incidence levels not explained by observed covariates [10]. As covariates in our study could not account for spatiotemporal heterogeneity, we introduced random effects in our fixed effect model to capture the heterogeneity in different components. The variances of random effects were 0.163 (0.086, 0.195), 1.3057 (0.8895, 1.8535), and 1.5493 (1.1173, 2.0107) in autoregressive component, spatiotemporal component, and endemic component, respectively. The estimates of random effects was summarized in Table A 5 (Additional file). As shown in Fig. 6, in terms of endemic component, the random effects of Panzhihua, Chengdu, Mianyang, and Dazhou are all greater than 1. In the autoregressive component, the random effect of Panzhihua, Aba, Luzhou, and Chengdu was greater than 1, while in the spatiotemporal component, only Zigong has a random effect greater than 1. The value above (or below)1 indicates that the average incidence rate in one area is higher (or lower) than in other areas [38]. Given the number of reported infections, this could be interpreted as a tendency to produce more or fewer cases in a particular area.

## Discussion

We analyzed the reported cases of scarlet fever in Sichuan from 2016 to 2019. Then explored the transmission dynamic rules based on three dimensions (i.e, endemic, epidemic within city and epidemic among cities). Moreover, we studied the potential ecological factors that could influence the spread of scarlet fever. Our study could improve epidemiological understanding of scarlet fever, depict the epidemiology status of the scarlet fever epidemic, capture the transmission pattern of scarlet fever, and provide clues for the future prevention and control of acute infectious diseases such as scarlet fever.

In our study, males outnumbered females in all patients, according to statistics, which is consistent with previous studies [20, 39]. This can be explained by the risk of exposure, for example, men have more outdoor activities and unhygienic behaviors than women [21]. Our study indicated over 50% of cases focused on the age group 4–6, which may be due to the lack of herd immunity to scarlet fever in the children aged 3–6 [39]. To prevent outbreaks in these vulnerable groups, there is an urgent need to strengthen the education on health and the environment of sterilization. Furthermore, we discovered that the major peak of incidence occurred in the summer, with a slight surge in the winter, which is typical of most respiratory diseases [15]. This suggests personal protection is primary during the peak season.

Our results are consistent with previous studies, the overall incidence of scarlet fever rises in recent years, and the southeast area has more cases than the northwest [40]. We also found that the incidence was randomly dispersed during 2016–2019 in Sichuan Province, this may be because the susceptible population is mainly children and teenagers, whose mobility in space are limited.

Although our results showed there were no spatial autocorrelation of scarlet fever in Sichuan in 2016–2019, we still conducted spatiotemporal analysis for three reasons: Firstly, previous simulation study domonstrated that significant covariate effect in multiple regression but not in univariate regression [41]. The multiple regression results only depends on univariate screening may be dramatically biased and misleading. Moreover, from a methodological perspective, the spatio-temporal model itself has the property of “borrowing strength” [42]. Our data contained both temporal and spatial information, and neglecting spatial information will lead to deviations. Secondly, combined with the actual situation of scarlet fever transmission in Sichuan Province, we found that transmission among cities had occurred. Finally, we compared the difference between including and not including the spatial component (Table A3 and Table A4 in additional file) in the spatiotemporal analysis. The results showed that the model with the spatial influence had a better fit with the same direction of estimates, so we considered it necessary to do spatiotemporal analysis.

As for the components of transmission of scarlet fever, endemic and autoregressive components accounted for major proportions of transmission in many areas in Sichuan province. This could be due to acute respiratory infections causing symptoms quickly, making relocation to another place unlikely [43]. Because scarlet fever is easily influenced by prior instances, it is critical to enhancing the early prediction and early warning of local cases, and it is recommended to detect and control outbreaks as early as possible to limit secondary cases. At the same time, controlling the epidemic of scarlet fever not only requires good local case management but also entails joint efforts with adjacent areas to reduce the spread of the epidemic.

The disparity in ecological conditions will finally cause health inequality [36]. In our study, we found that ecological factors could influence the transmission of scarlet fever, such as the school holidays could restrict the spread of scarlet fever. As school vacations play a significant role in the epidemiology of various infectious diseases [35], the incidence was lower on holidays because of fewer opportunities for contagiousness. Although researchers proved GDP was associated with the transmission of infectious diseases [37], the effect has not been quantified. Our finding suggests GDP could contribute to the transmission in the spatial aspect, which is probably because GDP can boost transmission by altering other factors such as population migration [33]. We also should consider GDP as an important factor when analyzing the potential drivers of acute infectious disease.

To improve the accuracy of our model, we incorporated random effects. If regions showed heterogeneity that cannot be explained by covariates, random effects may be able to capture this unexplained element of the model [44], especially in the multiple regions with the obvious distinction. One reason is that the incidence of scarlet fever in Sichuan province varies greatly among regions. For example, Chengdu has about one-third of the cases in the whole province, while Ganzi has no more than 10 cumulative cases in the 4 years. The other reason is that the ecological factors in our study cannot fully account for the heterogeneity among regions and components. Therefore, we used the random effect to explore heterogeneity in different components and regions. For the surveillance data of infectious diseases, the heterogeneity may be caused by missed diagnosis [45] and incomplete covariates.

Compare to traditional mathematical models, the EE model can study spatiotemporal dynamics from three dimensions: endemic, epidemic within city, and epidemic among cities. And also qualifies the effect of covariates from the above aspects. It could provide more information, such as the main transmission of scarlet fever in this paper—cases are predominantly affected by local past cases rather than cases in other areas and so on. In addition, it allowed random effects to capture more realism in the model.

There are several limitations in our study. As for the surveillance data, not all infected individuals seek medical care [45]. As for the model, although adding random effects can improve the representativeness of results [46], it could lead to more complexity in the model. Further simulation studies are needed to explore relatively optimal models with appropriate numbers of random effects.

With the prevalence of new respiratory infections and the global “resurgence” of historical infectious diseases such as scarlet fever, it is critical to optimize public health prevention and control strategies in the absence of targeted vaccines. Globally, acute respiratory infections are the major reason for morbidity and mortality in children under 5 [43]. Scarlet fever epidemics have historically occurred every 5–6 years, presumably due to the establishment of herd immunity in vulnerable groups and many influence factors [47]. There is currently no solid solution to eradicate acute respiratory infections. We must pay close attention to key populations and hotspots, as well as understand the elements that contribute to the spread of ARI. At the same time, a thorough understanding of the components of the transmission can help to strategically allocate resources to high-priority geographic areas and formulate specific public health interventions for varied regions. For example, if the epidemic is affected by cases in the previous period, early prediction and early warning of local epidemic need to be strengthened to reduce secondary cases. If the endemic component plays a significant role in transmission, it is necessary to enhance patient management and environmental disinfection to reduce the follow-up impact. To control the spread of the disease, not only do we need local case management but also entail joint prevention and control with neighboring regions can reduce the spread of the epidemic.

## Conclusion

By analyzing the data of scarlet fever from 2016 to 2019 in Sichuan Province, we explored the transmission pattern of this infectious disease from the endemic, temporal and spatial dimensions. Furthermore, we established the ecological factors that could influence transmission. Our work could benefit to optimize strategies for the prevention, detection, and management of scarlet fever and ARI.

In this paper, we took scarlet fever in Sichuan province as an example to explore the spatiotemporal transmission rules and potential ecological drivers. Firstly, We discovered that the majority of reported cases were transferred via local previous cases as well as external or indirect transmission through environmental sources. Secondly, we also found ecological factors could also influence the spread of acute respiratory infections such as scarlet fever. For example, school holidays could reduce transmission whereas GDP could boost the spread of scarlet fever. According to our findings, we should focus more on improving environmental disinfection and dealing with earlier occurrences. Because most ARIs lack viable vaccinations, public health initiatives remain critical in combating them. To maximize methods for the prevention, identification, and management of acute respiratory infectious illnesses, a focus on critical populations and hotspots is required, as well as an understanding of how ecological variables promote the spread of acute respiratory infectious diseases.

## Supplementary Information

**Additional file 1: Table A1.** The meaning and value of covariates. **Table A2.** Demographic characteristics of scarlet fever in Sichuan Province, 2016–2019. **Fig. A1.** Time series of scarlet fever counts in 21 cities (/prefectures) of Sichuan province from 2016 to 2019. **Figure A2.** The fitted values for 21 cities (/prefectures) in Sichuan Province during 2016–2019. **Fig. A3.** The estimated multiplicative effect of seasonality on the endemic mean. **Table A3.** Model selection and comparison. **Table A4.** Model without spatial term. **Table A5.** The estimates of random effects. **Fig. A4.** Spatial connectivity weights. **Fig. A5.** The matrix of cities showing the connectivity weights.

## Data Availability

The data that support the findings of this study are available from Sichuan Center for Disease Control and Prevention but restrictions apply to the availability of these data, which were used under license for the current study, and so are not publicly available. Data are however available from the authors upon reasonable request and with permission of Sichuan Center for Disease Control and Prevention.

## Abbreviations

ARI: Acute respiratory infection
COVID-19: Coronavirus disease 2019
GDP: Gross Domestic Product
AIC: Akaike information criterion
RPS: Ranked probability scores
logS: logS scores
RR: Relative risk
CI: Confidence interval
