# Supplementary material for: Spatiotemporal dynamics and potential ecological drivers of acute respiratory infectious diseases: an example of scarlet fever in Sichuan Province
Source: BMC Public Health. 2022 Nov 21;22:2139. doi: 10.1186/s12889-022-14469-y (PMC9680133; doi:10.1186/s12889-022-14469-y)

**Additional file 1**

# Supplementary Materials

# Table A1 The meaning and value of covariates

# Table A2 Demographic characteristics of scarlet fever in Sichuan Province, 2016–2019

# Figure A1 Time series of scarlet fever counts in 21 cities (/prefectures) of Sichuan province from 2016 to 2019

# Figure A2 The fitted values for 21 cities (/prefectures) in Sichuan Province during 2016–2019

# Figure A3 The estimated multiplicative effect of seasonality on the endemic mean

# Table A3 Model selection and comparison

# Table A4 Model without spatial term

# Table A5 The estimates of random effects

# Figure A4 Spatial connectivity weights

# Figure A5 The matrix of cities showing the connectivity weights

Table A1 The meaning and value of covariates

|  | Meaning | Range of values |
| --- | --- | --- |
| Population Density | Population density is a measurement of the number of people in an area. It is an average number. Population density is calculated by dividing the number of people by the area. Population density is usually shown as the number of people per 10,000 square kilometers. | 8-1157 (10,000 square kilometres) |
| School holidays | School holidays mainly refer to the winter and summer vacations in Sichuan province. | we set school holidays as a dummy variable, 0 denotes the school day, and 1 denotes the  summer and winter vacations. |
| GDP | Gross domestic product (GDP) is the total monetary or market value of all the finished goods and services produced within a region’s borders in a specific period. | 265.67-17012.65 (Trillion Yuan) |
| Number of health institutions | The number of health institutions per total population living in a designated area. | 1056-12121 |
| Number of kindergartens | The number of kindergartens per total population living in a designated area. | 192-2630 |
| Number of primary schools | The number of primary schools per total population living in a designated area. | 60-607 |
| Passenger-kilometers of highways | Passenger-Kilometers of Highways refers to the sum of actual delivery by a variety of means of transport highway with the corresponding number of passengers, which could reflect the mobility in space. | 60531-902156 (10,000 passenger-km） |

# Table A2 Demographic characteristics of scarlet fever in Sichuan Province, 2016–2019

|  |  |  | Year | | | | | | |  |
| --- | --- | --- | --- | --- | --- | --- | --- | --- | --- | --- |
|  | Total | | 2016 | | 2017 | | 2018 | | 2019 | |
|  | cases | incidence | cases | incidence | cases | incidence | cases | incidence | cases | incidence |
| Gender |  |  |  |  |  |  |  |  |  |  |
| female | 2,968 | 0.892 | 657 | 0.795 | 805 | 0.970 | 754 | 0.904 | 752 | 0.898 |
| male | 4,388 | 1.319 | 1,051 | 1.272 | 1,197 | 1.442 | 1,073 | 1.286 | 1,067 | 1.274 |
| Age (year) |  |  |  |  |  |  |  |  |  |  |
| 0-3 | 1,287 | 0.387 | 306 | 0.370 | 356 | 0.429 | 298 | 0.357 | 327 | 0.390 |
| 4-6 | 3,890 | 1.169 | 916 | 1.109 | 1,096 | 1.320 | 1,003 | 1.202 | 875 | 1.045 |
| 7-9 | 1,670 | 0.502 | 390 | 0.472 | 453 | 0.546 | 399 | 0.478 | 428 | 0.511 |
| 10-12 | 296 | 0.089 | 64 | 0.077 | 72 | 0.087 | 86 | 0.103 | 74 | 0.088 |
| 13-15 | 106 | 0.032 | 15 | 0.018 | 16 | 0.019 | 19 | 0.023 | 56 | 0.067 |
| 16-59 | 107 | 0.032 | 17 | 0.021 | 9 | 0.011 | 22 | 0.026 | 59 | 0.070 |
| Source of cases | |  |  |  |  |  |  |  |  |  |
| Other province | 57 | 0.017 | 11 | 0.013 | 16 | 0.019 | 11 | 0.013 | 19 | 0.023 |
| Foreign country | 1 | 0.000 | 1 | 0.001 | 0 | 0.000 | 0 | 0.000 | 0 | 0.000 |
| The same city | 7,160 | 2.151 | 1,675 | 2.027 | 1,933 | 2.328 | 1,728 | 2.072 | 1,770 | 2.113 |
| Other cities | 138 | 0.041 | 21 | 0.025 | 53 | 0.064 | 34 | 0.041 | 30 | 0.036 |
| Total | 7,356 | 2.210 | 1,708 | 2.067 | 2,002 | 2.411 | 1,827 | 2.190 | 1,819 | 2.172 |

Note: incidence is the average annual incidence rate (per 100,000 population)

# Figure A1 Time series of scarlet fever counts in 21 cities (/prefectures) of Sichuan province from 2016 to 2019

**
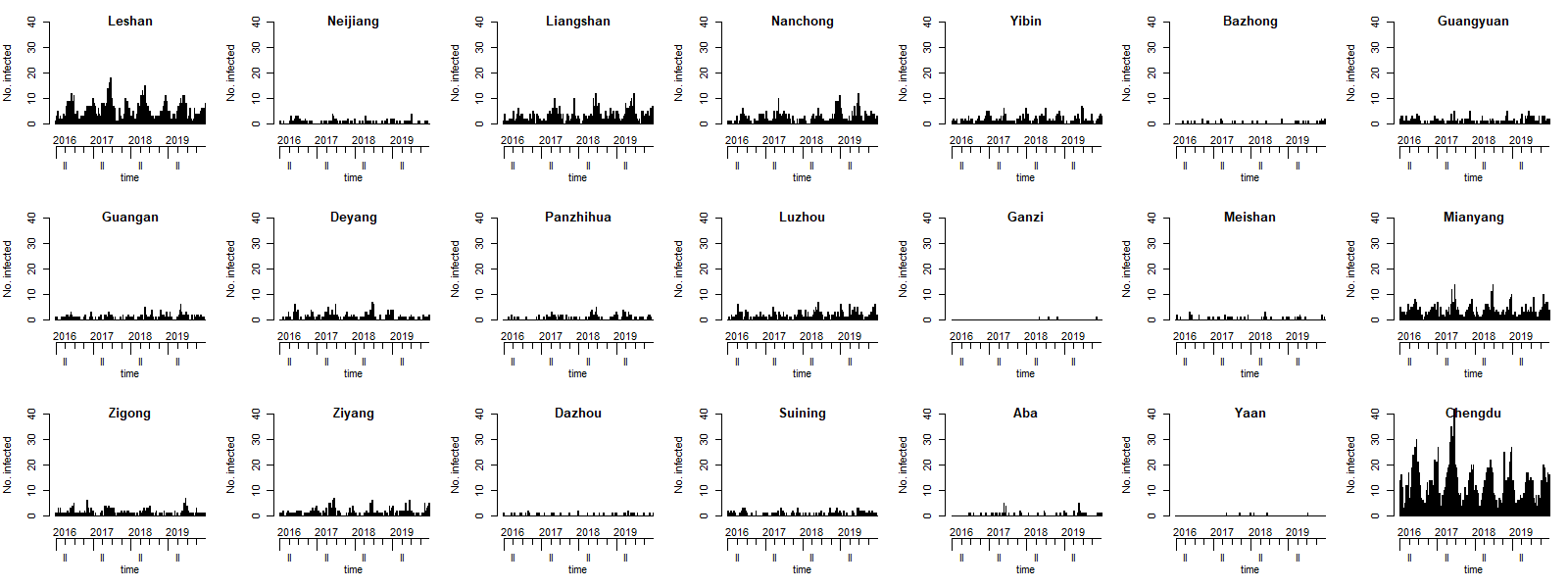
**

# Figure A2 The fitted values for 21 cities (/prefectures) in Sichuan province during 2016-2019


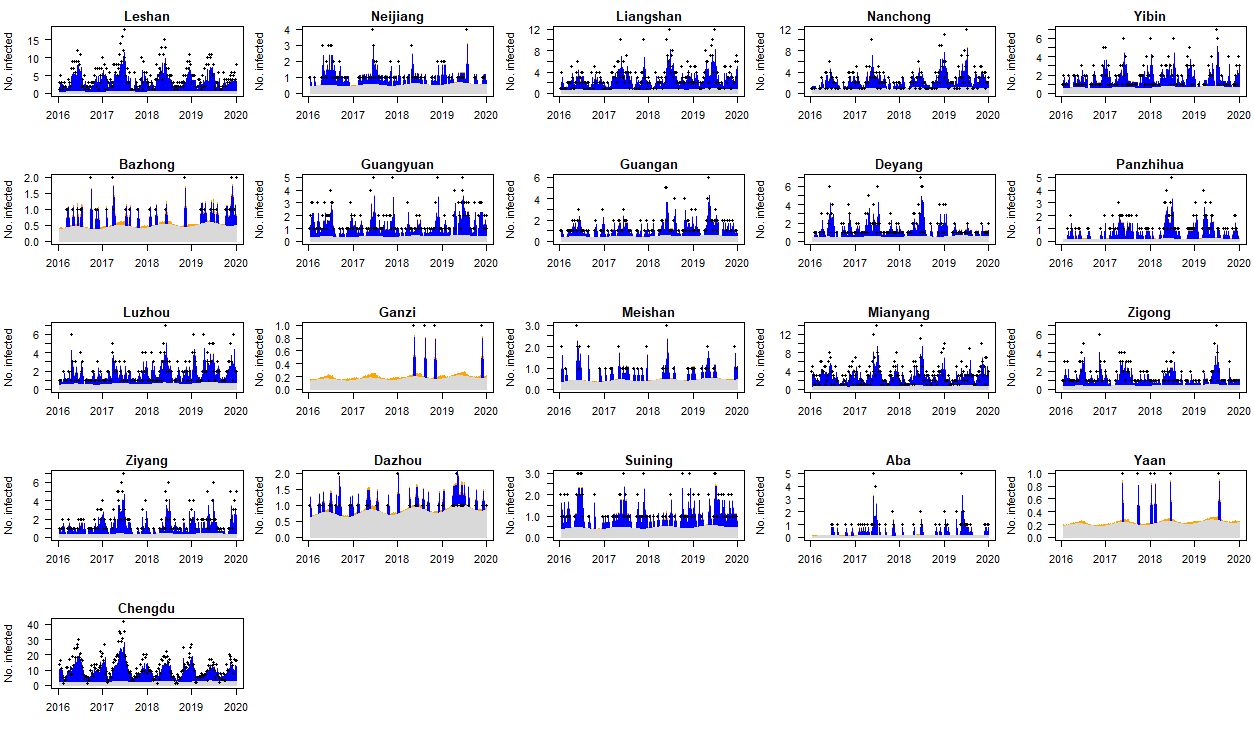


# Figure A3. The estimated multiplicative effect of seasonality on the endemic mean


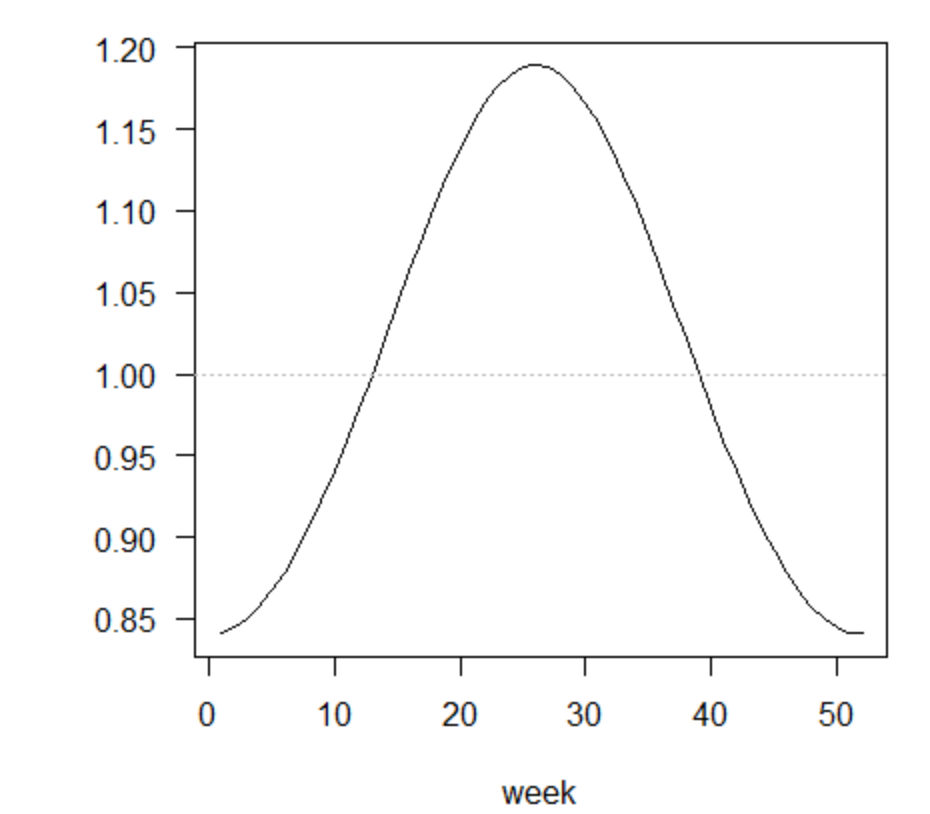


Table A3 Model selection and comparison

|  |  | Model 1 | |  | Model 2 | |  | Model 3 | |  | Model 4 | |  | Model 5 | | Model 6 | (with random effect) | | |
| --- | --- | --- | --- | --- | --- | --- | --- | --- | --- | --- | --- | --- | --- | --- | --- | --- | --- | --- | --- |
| Parameter | RR | 95%CI | | RR | 95%CI | | RR | 95%CI | | RR | 95%CI | | RR | 95%CI | | RR | 95%CI | | *P value* |
|  |  | lower | upper |  | lower | upper |  | lower | upper |  | lower | upper |  | lower | upper |  | lower | upper |  |
| Epidemic within city (autoregressive) | | | | | | | | | | | | | | | | | | | |
| Intercept | **0.5383** | 0.3803 | 0.7620 | 0.7057 | 0.4821 | 1.0331 | **0.3376** | 0.1854 | 0.6149 | 0.4678 | 0.1621 | 1.3499 | 0.5022 | 0.1529 | 1.6489 | 0.1588 | 0.0034 | 7.4853 | 0.349 |
| Density | 1.0444 | 0.9858 | 1.1065 | 0.9853 | 0.9238 | 1.0510 | **0.7475** | 0.6828 | 0.8184 | **0.7528** | 0.6871 | 0.8247 | **0.6673** | 0.579 | 0.7692 | 0.8162 | 0.5192 | 1.2831 | 0.379 |
| School holidays |  |  |  | **0.6666** | 0.5608 | 0.7924 | **0.6429** | 0.5408 | 0.7643 | **0.6198** | 0.5098 | 0.7535 | **0.6325** | 0.5124 | 0.7809 | **0.4712** | 0.2776 | 0.7999 | 0.005 |
| GDP |  |  |  |  |  |  | **1.3327** | 1.1965 | 1.4844 | **1.3483** | 1.1657 | 1.5595 | **1.1278** | 0.9627 | 1.3212 | 1.6331 | 0.9146 | 2.916 | 0.097 |
| number of health institutions |  |  |  |  |  |  |  |  |  | 0.9484 | 0.7888 | 1.1403 | 0.7983 | 0.569 | 1.1201 | 0.8328 | 0.2562 | 2.7073 | 0.761 |
| number of kindergartens |  |  |  |  |  |  |  |  |  |  |  |  | 2.1945 | 1.6516 | 2.916 | 1.0954 | 0.4324 | 2.7752 | 0.848 |
| number of primary schools |  |  |  |  |  |  |  |  |  |  |  |  | **0.7254** | 0.5344 | 0.9848 | 0.7951 | 0.3146 | 2.0093 | 0.628 |
| Epidemic among cities (spatiotemporal) | | | | | | | | | | | | | | | | | | | |
| Intercept | **0.0016** | 0.0001 | 0.0181 | **0.0001** | 0.0001 | 0.6598 | **0.0002** | 0.0001 | 0.0003 | 0.0001 | 0.0001 | 0.0007 | **0.0049** | 7.00E-04 | 0.0342 | 0.0024 | 0.0001 | 2.5915 | 0.091 |
| Density | **1.9458** | 1.3258 | 2.8557 | 2.7089 | 0.7216 | 10.1694 | 1.0114 | 0.9293 | 1.1007 | 0.9806 | 0.8398 | 1.1450 | **1.2406** | 1.0412 | 1.4781 | 1.1721 | 0.6524 | 2.1058 | 0.595 |
| GDP |  |  |  |  |  |  | **2.8354** | 2.5248 | 3.1842 | **3.2734** | 2.7182 | 3.9420 | **2.7515** | 2.1115 | 3.5854 | **2.6898** | 1.1516 | 6.2822 | 0.022 |
| Passengers |  |  |  |  |  |  |  |  |  |  |  |  | **0.6769** | 0.538 | 0.8516 | 0.7528 | 0.4971 | 1.14 | 0.18 |
| Endemic | | | | | | | | | | | | | | | | | | | |
| Intercept | 1.0018 | 1.0005 | 1.0031 | **1.0014** | 1.0002 | 1.0026 | 0.9979 | 0.9909 | 1.0048 | 0.9990 | 0.9969 | 1.0010 | 0.9986 | 0.9965 | 1.0006 | 0.9965 | 0.9929 | 1.0001 | 0.053 |
| School holidays |  |  |  | **0.6693** | 0.5554 | 0.8065 | 313.2752 | 0.4519 | 217172.6761 | **0.6841** | 0.5098 | 0.9181 | **0.6129** | 0.4526 | 0.83 | **0.5299** | 0.3883 | 0.7233 | <0.001 |
| number of kindergartens |  |  |  |  |  |  |  |  |  |  |  |  | 1.1684 | 0.9231 | 1.4789 | 2.144 | 0.6288 | 7.3111 | 0.223 |
| number of primary schools |  |  |  |  |  |  |  |  |  |  |  |  | **2.9397** | 2.0369 | 4.2427 | 0.4996 | 0.178 | 1.4021 | 0.188 |
| AIC | 11903 |  |  | 11837.21 |  |  | 11743.17 |  |  | 11670.93 |  |  | 11604.41 |  |  | - |  |  |  |
| logs | 1.543 |  |  | 1.526 |  |  | 1.534 |  |  | 1.51 |  |  | 1.516 |  |  | 1.424 |  |  |  |
| rps | 0.809 |  |  | 0.782 |  |  | 0.77 |  |  | 0.753 |  |  | 0.741 |  |  | 0.653 |  |  |  |
| Note: Sequential fixed model building from intercept-only model (model 1) to the final model (model 5) and the optimal model(model 6) introduced ramdom effects based on model 5; Statistically significant explanatory factors are in bold | | | | | | | | | | | | | | | | | | | |

# Table A4 Model without spatial term

|  |  | Model 1 | |  | Model 2 | |  | Model 3 | |  | Model 4 | |  | Model 5 | | Model 6 | (with random effect) | | |
| --- | --- | --- | --- | --- | --- | --- | --- | --- | --- | --- | --- | --- | --- | --- | --- | --- | --- | --- | --- |
| Parameter | RR | 95%CI | | RR | 95%CI | | RR | 95%CI | | RR | 95%CI | | RR | 95%CI | | RR | 95%CI | | *P* value |
|  |  | lower | upper |  | lower | upper |  | lower | upper |  | lower | upper |  | lower | upper |  | lower | upper |  |
| Epidemic within city (autoregressive) | | | | | | | | | | | | | | | | | | | |
| Intercept | 0.6342 | 0.4417 | 0.9106 | 0.6515 | 0.4531 | 0.9368 | 0.2037 | 0.1341 | 0.3095 | 0.4518 | 0.1812 | 1.1265 | 0.9479 | 0.3091 | 2.9072 | 0.0711 | 0.003 | 1.7088 |  |
| Density | 0.9935 | 0.9348 | 1.056 | 0.9968 | 0.9378 | 1.0595 | 0.7665 | 0.7061 | 0.8321 | 0.7576 | 0.6967 | 0.8237 | 0.7318 | 0.6424 | 0.8337 | 0.8682 | 0.6059 | 1.2439 | 0.441 |
| School holidays |  |  |  | 0.6616 | 0.5557 | 0.7877 | 0.6535 | 0.5501 | 0.7765 | 0.6563 | 0.5521 | 0.7801 | 0.6453 | 0.5384 | 0.7734 | 0.5434 | 0.4026 | 0.7334 | <0.001 |
| GDP |  |  |  |  |  |  | 1.4177 | 1.3127 | 1.5312 | 1.5472 | 1.3718 | 1.745 | 1.3585 | 1.174 | 1.5719 | 1.3284 | 0.8531 | 2.0687 | 0.209 |
| number of health institutions |  |  |  |  |  |  |  |  |  | 0.846 | 0.712 | 1.0054 | 0.6035 | 0.4296 | 0.8478 | 1.0616 | 0.4405 | 2.5581 | 0.894 |
| number of kindergartens |  |  |  |  |  |  |  |  |  |  |  |  | 1.4875 | 1.1582 | 1.9105 | 1.177 | 0.5786 | 2.3942 | 0.653 |
| number of primary schools |  |  |  |  |  |  |  |  |  |  |  |  | 1.1165 | 0.8583 | 1.4525 | 0.7709 | 0.3649 | 1.6286 | 0.495 |
| Endemic | | | | | | | | | | | | | | | | | | | |
| Intercept | 1.0016 | 1.0005 | 1.0026 | 1.0013 | 1.0003 | 1.0023 | 1.0009 | 0.9999 | 1.0019 | 1.0009 | 0.9999 | 1.0019 | 1.0009 | 0.9999 | 1.0019 | 0.0105 | 2.00E-04 | 0.6902 | <0.001 |
| School holidays |  |  |  | 0.6518 | 0.5544 | 0.7664 | 0.6387 | 0.5438 | 0.7501 | 0.6363 | 0.542 | 0.7469 | 0.6291 | 0.5375 | 0.7364 | 0.5854 | 0.5125 | 0.6687 | <0.001 |
| number of kindergartens |  |  |  |  |  |  |  |  |  |  |  |  | 1.4127 | 1.2057 | 1.6553 | 0.7658 | 0.4141 | 1.416 | 0.395 |
| number of primary schools |  |  |  |  |  |  |  |  |  |  |  |  | 0.8081 | 0.6488 | 1.0064 | 0.97 | 0.4772 | 1.9721 | 0.933 |
| AIC | 11933.73 |  |  | 11846.7 |  |  | 11764.91 |  |  | 11763.3 |  |  | 11730.46 |  |  | - |  |  |  |
| logs | 1.528 |  |  | 1.514 |  |  | 1.503 |  |  | 1.507 |  |  | 1.508 |  |  | 1.448 |  |  |  |
| rps | 0.8 |  |  | 0.774 |  |  | 0.76 |  |  | 0.763 |  |  | 0.756 |  |  | 0.678 |  |  |  |

Note: Sequential fixed model building from intercept-only model (model 1) to the final model (model 5) and the optimal model(model 6) introduced ramdom effects based on model 5;

Table A5 The estimates of random effects

| Random effects | RR | 95%CI | | *P* value |
| --- | --- | --- | --- | --- |
|  |  | upper | lower |  |
| Epidemic within city (autoregressive) | | | | |
| Leshan | 1.1716 | 0.6735 | 2.038 | 0.575 |
| Neijiang | 0.7222 | 0.3669 | 1.4214 | 0.346 |
| Liangshan | 1.47 | 0.7416 | 2.914 | 0.27 |
| Nanchong | 1.1315 | 0.5664 | 2.2605 | 0.726 |
| Yibin | 0.9039 | 0.5068 | 1.6119 | 0.732 |
| Bazhong | 0.9471 | 0.4685 | 1.9148 | 0.88 |
| Guangyuan | 0.8737 | 0.4511 | 1.6925 | 0.689 |
| Guang’an | 1.1956 | 0.6391 | 2.2369 | 0.576 |
| Deyang | 0.9716 | 0.5067 | 1.8631 | 0.931 |
| Panzhihua | 1.4847 | 0.742 | 2.9706 | 0.264 |
| Luzhou | 0.9617 | 0.5301 | 1.7447 | 0.898 |
| Ganzi | 0.9055 | 0.4228 | 1.9394 | 0.798 |
| Meishan | 0.8418 | 0.4365 | 1.6234 | 0.607 |
| Mianyang | 0.8122 | 0.4519 | 1.4596 | 0.487 |
| Zigong | 0.6872 | 0.3657 | 1.2916 | 0.244 |
| Ziyang | 1.2145 | 0.6215 | 2.3733 | 0.57 |
| Dazhou | 0.7877 | 0.4144 | 1.4972 | 0.466 |
| Suining | 1.1131 | 0.5827 | 2.1263 | 0.746 |
| Aba | 0.9799 | 0.4759 | 2.0176 | 0.956 |
| Ya’an | 0.87 | 0.4131 | 1.8323 | 0.714 |
| Chengdu | 1.4828 | 0.7389 | 2.9756 | 0.268 |
| Epidemic among cities (spatiotemporal) | | | | |
| Leshan | 3.9366 | 1.8292 | 8.4721 | <0.001 |
| Neijiang | 0.5708 | 0.2014 | 1.6177 | 0.291 |
| Liangshan | 3.1687 | 1.1534 | 8.7054 | 0.025 |
| Nanchong | 2.8815 | 1.4418 | 5.7587 | 0.003 |
| Yibin | 0.842 | 0.373 | 1.9009 | 0.679 |
| Bazhong | 0.4154 | 0.1215 | 1.4205 | 0.161 |
| Guangyuan | 1.9949 | 0.9107 | 4.3699 | 0.084 |
| Guang’an | 1.3016 | 0.5486 | 3.0883 | 0.55 |
| Deyang | 0.7272 | 0.3059 | 1.7285 | 0.471 |
| Panzhihua | 0.7028 | 0.2362 | 2.0916 | 0.526 |
| Luzhou | 1.9499 | 0.9359 | 4.0622 | 0.075 |
| Ganzi | 0.2899 | 0.0583 | 1.441 | 0.13 |
| Meishan | 0.3138 | 0.1224 | 0.8047 | 0.016 |
| Mianyang | 3.2599 | 1.2948 | 8.2074 | 0.012 |
| Zigong | 0.9483 | 0.3591 | 2.5041 | 0.915 |
| Ziyang | 3.4968 | 1.2991 | 9.4122 | 0.013 |
| Dazhou | 0.2117 | 0.0825 | 0.5435 | 0.001 |
| Suining | 0.7615 | 0.2998 | 1.9341 | 0.567 |
| Aba | 3.3242 | 0.7833 | 14.1078 | 0.103 |
| Ya’an | 0.114 | 0.0354 | 0.3667 | <0.001 |
| Chengdu | 0.8963 | 0.1414 | 5.6817 | 0.908 |
| Endemic | | | | |
| Leshan | 7.0567 | 3.335 | 14.9316 | <0.001 |
| Neijiang | 1.075 | 0.4607 | 2.5082 | 0.867 |
| Liangshan | 8.2206 | 1.7757 | 38.0578 | 0.007 |
| Nanchong | 0.3259 | 0.0794 | 1.3384 | 0.12 |
| Yibin | 1.5341 | 0.6278 | 3.7492 | 0.348 |
| Bazhong | 0.6912 | 0.2034 | 2.3488 | 0.554 |
| Guangyuan | 3.4318 | 1.1217 | 10.4994 | 0.031 |
| Guang’an | 0.4538 | 0.1183 | 1.7406 | 0.249 |
| Deyang | 1.9802 | 0.6491 | 6.0412 | 0.23 |
| Panzhihua | 1.6616 | 0.3666 | 7.5306 | 0.51 |
| Luzhou | 1.062 | 0.3652 | 3.088 | 0.912 |
| Ganzi | 0.2294 | 0.046 | 1.1453 | 0.073 |
| Meishan | 0.4557 | 0.1402 | 1.4812 | 0.191 |
| Mianyang | 2.2613 | 0.7521 | 6.7991 | 0.146 |
| Zigong | 2.0477 | 0.748 | 5.6061 | 0.163 |
| Ziyang | 0.3795 | 0.08 | 1.8 | 0.222 |
| Dazhou | 0.1997 | 0.0667 | 0.5986 | 0.004 |
| Suining | 0.8261 | 0.2757 | 2.4758 | 0.733 |
| Aba | 0.597 | 0.0909 | 3.9199 | 0.591 |
| Ya’an | 0.2164 | 0.0502 | 0.9336 | 0.04 |
| Chengdu | 2.1773 | 0.4469 | 10.6068 | 0.335 |

Figure A4 Spatial connectivity weights


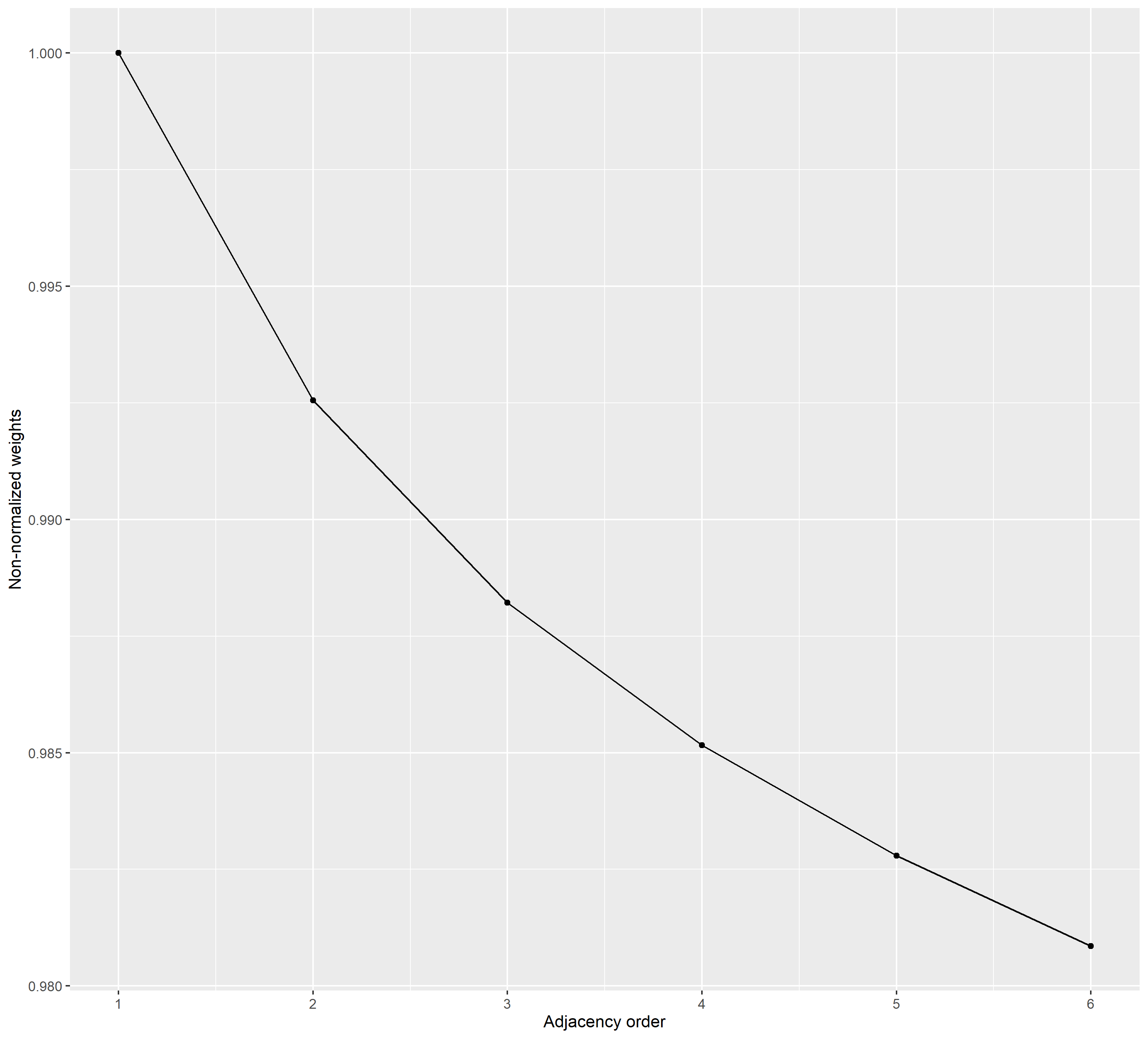


Figure A5 The matrix of cities showing the connectivity weights


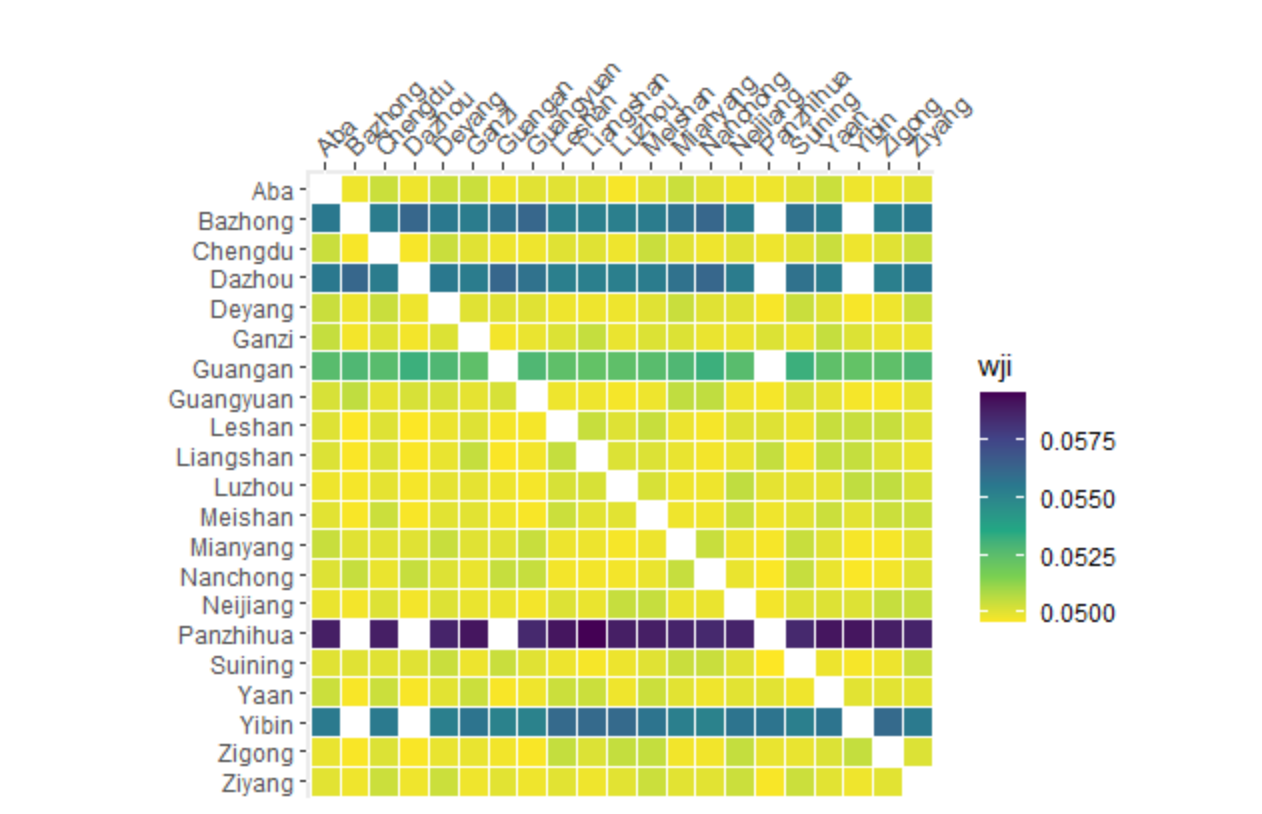

Supplement: Supplementary file 1 — Additional file 1: Table A1. The meaning and value of covariates. Table A2. Demographic characteristics of scarlet fever in Sichuan Province, 2016–2019. Fig. A1. Time series of scarlet fever counts in 21 cities (/prefectures) of Sichuan province from 2016 to 2019. Figure A2. The fitted values for 21 cities (/prefectures) in Sichuan Province during 2016–2019. Fig. A3. The estimated multiplicative effect of seasonality on the endemic mean. Table A3. Model selection and comparison. Table A4. Model without spatial term. Table A5. The estimates of random effects. Fig. A4. Spatial connectivity weights. Fig. A5. The matrix of cities showing the connectivity weights. [file 12889_2022_14469_MOESM1_ESM.docx]
